# Mainstream Preschool Teachers’ Skills at Identifying and Referring Children with Autism Spectrum Disorder (ASD)

**DOI:** 10.3390/ijerph17124284

**Published:** 2020-06-16

**Authors:** Sahar Mohammed Taresh, Nor Aniza Ahmad, Samsilah Roslan, Aini Marina Ma’rof, Sumaia Mohammed Zaid

**Affiliations:** 1 Department of Foundations of Education, Faculty of Educational Studies, University Putra Malaysia, Serdang 43400, Malaysia; sahartaresh@yahoo.com (S.M.T.); samsilah@gmail.com (S.R.); ainimarina@upm.edu.my (A.M.M.); 2 Department of Psychology, Sana’a University, Sana’a 1247, Yemen; sumaiamohammed@hotmail.com

**Keywords:** autism spectrum disorder, identify, identifications skills, refer, preschool teachers

## Abstract

*Background:* Early intervention will help children with autism spectrum disorder (ASD) to attain early learning reinforcement. This study focuses on exploring the ability of preschool teachers to identify children with ASD and their referral decision-making process. *Method:* This is a mixed-method study (qualitative and quantitative methods) involving 20 respondents. The qualitative study is based on an open question case study, while the quantitative study consists of questionnaire with demographic variables to identify the effect of the demographic variables on the preschool teachers’ ability to identify children with ASD. *Sample:* The sample was selected via convenience sampling among mainstream preschool teachers. The data was analyzed using SPSS software and thematic analysis. *Results:* The findings show that preschool teachers did not have skills at identifying children with ASD, and the majority of them labelled children with ASD as spoilt or hyperactive children. They also viewed children with ASD as having other disorders such as attention deficit hyperactivity disorder (ADHD) or communication disorders such as introversion. Moreover, preschool teachers expressed that the reason for the child’s behaviour could be due to the parents’ inability to properly educate their child. Additionally, the demographic variables of the preschool teachers, such as age, education level and teaching experience, were found not to affect their ASD identification skills. *Conclusion:* Preschool teachers need to improve their skills in identifying ASD among children via training.

## 1. Introduction

The Republic of Yemen’s decision in 1991 marked the first legislation in Yemen for the care and rehabilitation of children with disabilities. Then, in 1999, the Higher Committee for the Care of Handicapped Rehabilitation was established and the Handicapped Care and Rehabilitation Fund was launched. Unfortunately, the Handicapped Care and Rehabilitation Fund considered autism spectrum disorder (ASD) as one of the disability categories. In their strategy for the years 2004–2018, the first goal was to change society’s view towards children with disabilities by raising the level of awareness of their rights and capabilities. During that period, Yemen was rapidly improving and heading towards urbanization, while suffering from a deteriorating economy. According to The United Nations Human Development Report in 2007, Yemen was estimated as one of the poorest countries in the world. Moreover, it was ranked 140th out of 182 countries [1]. Low admission to education became an essential poverty-related issue in Yemen. According to the World Bank, 87% of the poor in Yemen are illiterate or have not completed primary school and the ongoing war contributed to the lack of education and poor health [1]. There is also a lack of ASD centers, with the exception of those in the main cities like Sana’a, Taiz and Aden. In addition, there is a severe scarcity of child mental health professionals [2]. All of these factors have resulted in the loss of actual recordings of ASD cases. It is assumed that there are a large number of cases that have not been diagnosed [3,4]. 

On the other hand, Al-Zaalah et al. further mentioned that the early diagnosis of ASD is still limited in Arab countries such as Saudi Arabia, which is a neighboring country of Yemen. Early identification and intervention are important in order to increase children’s outcomes, especially among preschool children aged three to five years [5,6,7]. However, many ASD cases are identified late. During the preschool stage, early warning signs become clear evidence of delays in development, incapacity or potential disorders because teachers are more likely to distinguish the characteristics of ASDs than parents [8,9]. Early identification and intervention of mental health problems in childhood is essential to improve developmental routes and reduce the severity of emotional and behavioural disorders. Thus, preschool teachers can play an important role in early problem recognition. This role is particularly impactful in developing countries with limited mental health care resources. However, Yemeni preschool teachers have a lack of consideration for health and mental health needs [10,11]. Furthermore, Yemeni preschool teachers’ knowledge about mental health varies dramatically [12].

The currently increasing numbers of children with autism spectrum disorder (ASD) indicate that ASD is now more common than cancer, paediatric Acquired Immune Deficiency Syndrome (AIDS) combined, and juvenile diabetes [13]. The high percentage of ASD among children requires preschool teachers to exhibit high standards of work to meet the children’s abnormal development and to identify them from an early stage followed by their referral to diagnosis services [14,15,16].

The symptoms of behavioural disorders such as autism, attention deficit hyperactivity disorder (ADHD), depression, and anxiety can be detected in children before they begin elementary school [17,18,19]. With the early identification of children’s developmental and learning problems and prompt referrals for assessment, the children’s conditions, development and learning needs will be better understood and cared for. Moreover, researchers agree that the early identification of children with ASD and subsequent treatment can ultimately lead to better outcomes and quality of life [20,21,22]. Furthermore, several studies have confirmed that the early detection of such problems and referrals to appropriate services could alter negative trajectories and prevent more serious problems from developing [23,24]. In line with these findings, the US Interagency Autism Coordinating Committee declared two major objectives: (a) to reduce the disparities in early access to detection and services and (b) to improve models for detecting ASD [25].

However, the reasons for diagnoses at a late median age have not been well researched. Beyond parent and family factors (e.g., socioeconomic status, cultural and language diversity), community resources and policies could also play a role [26,27]. To identify and remove barriers to earlier diagnosis, one must first understand what happens when a child comes into contact with early childhood education preschool teachers who may have a gatekeeping role. Therefore, an integral part of a teacher’s role is to be professionally concerned towards children with behavioural difficulties [28], an example of which is ASD. Therefore, teachers (including preschool teachers (PST)) have the opportunity to play a key role in the early identification and referral of children’s developmental, behavioural, and emotional problems [29,30]. Therefore, schools and preschools are ideal settings for screening and identification, because they both have similar services and service access, and stigma and barriers against formal developmental delay services. Preschool teachers can often be more objective observers of children’s development compared to parents [31]. Preschool teachers also have the advantage of being able to assess a child’s functioning in comparison to a naturalistic developmental setting where the child may display a range of normative behaviours [32]. 

A preschool teacher has high chances of detecting the above disorders in children and can subsequently identify the children’s situation and refer them for appropriate assessment, which, in turn, may lead to early intervention services [29]. Nevertheless, several studies have mentioned that preschool teachers might perceive numerous obstacles when identifying and referring these children for assessment [29,33,34]. These factors could fall under the personal characteristics of the preschool teachers, such as their knowledge, attitude, feelings, skills, and perceptions of children with ASD [29,33]. Furthermore, the teachers’ motivation to detect this type of disorder and then refer them could also be an obstacle [35]. Previous research has also confirmed that the teachers’ perceptions of the special service and resource availability could influence their referral decisions [6]. Furthermore, some teachers do not want to overload their school counsellor colleagues with their doubts about a child’s disorder [36].

Additionally, school counsellors perceive that they are already overloaded with assessment cases and do not have time for counselling or intervention work [37]. Furthermore, another study confirmed that students with less observable internalising concerns were significantly less likely to report school and community-based intervention services than students with externalising concerns, and these students would likely be perceived as no different than students with internalised and externalised behaviours that are rated in the normal range [38]. This phenomenon has been referred to as the “squeaky wheel”, in which children who disrupt the classroom or learning environment due to more noticeable educational and behavioural problems are more likely to receive services than students with less observable or disruptive difficulties [39]. Meanwhile, another study mentioned that despite the prevalence of ASD cases, these disorders are often detected late because of the lack of overt or observable symptoms, such as disrupting class and/or violating school rules [38]. Another study in Ethiopia confirmed that the reason for late detection was the limited identification and referral skills among preschool teachers regarding student developmental delays [40,41]. Furthermore, in developing countries, preschool teachers with more limited developmental delay training resources would likely be even less well equipped for this role. However, cross-cultural studies have revealed that teachers can recognise symptoms of behavioural problems such as ASD, but their actions, based on their ability to recognise, may be influenced by a variety of factors including the child’s characteristics and cultural expectations [42]. Another factor, in this case, is a shortage of knowledge and skills in handling preschoolers with challenging behaviour, with teachers citing that they lacked training in this area [43]. In other words, the preschool teachers’ knowledge and skills in identifying challenging behaviour in preschoolers may impact their identification and referral decisions later on [5,6,44,45]. Moreover, studies have indicated that only 8% of teachers would assess ASD among children using a formal measure while most would use informal assessments [46,47]. As a result, recent studies are increasingly insisting on educating teachers to recognise the early signs of ASD to enable them to identify early symptoms of ASD and to refer children with ASD to get professional assistance from the early stages of childhood [48]. Studies involving teachers have also found that an absence of knowledge and skills in handling children with challenging behaviour is an obstacle to ASD detection and intervention.

From our review of the literature, we found that preschool teachers have been questioned on the factors they felt impacted their referral decision of children with behavioural difficulties [33]. The teachers noted that the identification of ASD and their referral of suspected children with ASD as being more important to them than any other issue [43]. This result means that preschool teachers have the motivation to handle this type of disorder, but they need further education on how to identify the disorder.

Rosenbaum [49] tried to better understand pre-referral perceptions and decision factors among 346 teachers and found that the decision factors cited most often were play, social interaction/engagement, and verbal behaviour, but none were cited by a clear majority. Some important early signs were infrequently mentioned, namely absence of joint attention, social reciprocity, and gestures. The accuracy of referral decisions was similar across disciplines, indicating that professionals from broad-ranging fields are capable of identifying early signs of autism. Therefore, autism training for early childhood professionals should emphasise on the importance of early signs involving significant absences of behaviours, such as low joint attention, gestures, and social reciprocity in addition to odd social, verbal, and play behaviours. Such targeted training may encourage earlier referrals if autism is suspected in young children. Splett [38] examined the ability of teachers (*n* = 153) to accurately identify mental health concerns among elementary children using vignette scenarios depicting children with severe and moderate externalised or internalised behavioural problems. The findings indicated that teachers could accurately identify students with severe externalised or internalised problems. However, they were less accurate and less likely to think the students with moderate or subclinical symptoms required services.

Meanwhile, Semmel [50] reviewed recent empirical findings, which failed to support currently mandated identification procedures. The study also discussed the teachers’ decisions to refer children for special education and proposed reasonable options for rethinking the basic assumptions of special education for the mildly handicapped. Specifically, the study recommended designing (a) new identification procedures at the school site level and (b) a program of research aimed at investigating teachers’ referral decisions.

Moreover, Gabrielsen [49] aimed to better understand pre-referral perceptions and decision factors. The study provided multiple video clips from early comprehensive autism evaluations as prompts, and then asked early childhood clinicians and educators (*n* = 346) to make decisions for autism referral, followed by identifying the factors considered when they are making the decisions. 

In previous literatures, the preschool teachers’ shortage of knowledge and skills in handling behavioural difficulties were found to be the main factors affecting their ability to identify children with ASD [5,43].

In contrast, although there are many possible reasons for delayed evaluations, the referral decision process itself is not clearly defined nor understood. In particular, little is known about the effect of the teachers’ perceptions of early childhood symptoms of ASD and their decision criteria to ultimately refer the child for comprehensive diagnostic assessment [49]. A comprehensive review of the literature showed a significant gap in the current understanding of the factors that early childhood professionals consider when making decisions on whether or not to refer a child for comprehensive autism evaluation [51]. However, some studies have confirmed that the actual barriers to early identification and the referral decision-making process have not been extensively explored [49]. Unfortunately, in Yemen, children with ASD often receive a late diagnosis [3], which may have a negative impact on their development. Therefore, this study explores Yemeni preschool teachers’ ability to detect a child with abnormal behaviour in their class. Furthermore, this study focuses on exploring preschool teachers’ ability to identify children with ASD and their referral decision-making process. Accordingly, the objectives of this study are:

1. to determine preschool teachers’ ability to detect behaviour problems in the children presented in each of the video cases;
2. to explain preschool teachers’ ability to recognise a child with ASD;
3. to determine preschool teachers’ ability to make decisions regarding autism risk and the types of observations that could be regarded as “deciding” factors;
4. to determine preschool teachers’ ability to identify children with ASD through two videos (video 1 and video 2), based on their demographic variables (age, level of education, teaching experience).

## 2. Method

### 2.1. Design

This study adopted a mixed-method approach in which a case study was prepared for the qualitative study, and a demographic questionnaire for preschool teachers was developed for the quantitative study.

### 2.2. Participants and Process

The current study is conducted in the Republic of Yemen, specifically in the city of Taiz, which is one of the most densely populated cities in Yemen. Taiz is also known as the capital of culture of Yemen. It consists of three towns (Al Muzaffar, Al Kahera and Salaa). According to the 2014 statistics, there are 125 government preschools, 230 private preschools and a total of 355 preschool teachers in Taiz. The participants of this study consisted of mainstream preschool teachers. The data were collected from 20 participants from five different schools. Each participant used a pencil-and-paper protocol to answer the questions and to record their judgment of the symptoms of disorders, which they observed from two supplied video cases. The questionnaire designed in this study was not related to the teachers’ level of knowledge regarding early autism symptoms, so any bias related to prior knowledge levels was removed. Two video cases (ADHD; ASD) were presented to the participants to reduce knowledge bias in determining whether or not a child had ASD. This study precisely looked at how preschool teachers detected behaviour problems in the children presented in each of the video cases. and how they determined the child had ASD, and also how they made the decisions regarding the autism risk and the types of observations that could be regarded as “deciding” factors. Furthermore, this study looked at how demographic variables (age, level of education, teaching experience) affect preschool teachers’ ability when identifying children with ASD.

Using the video cases, the preschool teachers were exposed to the responses in those cases. In this research, official permission was obtained to engage the participants during school hours. The participants were asked to use a pen and paper to answer the questionnaire. The participants were asked to focus on the videos and then to answer the questions provided. On average, the participants took 30 min to complete the questionnaire. There were four questions to be answered after each video session. The data was analysed using SPSS software version 22 using descriptive and inferential analyses.

### 2.3. Instrument

The questionnaire in this study consisted of two parts. Part 1 consists of the participants’ demographic variables:

- What is your level of education? (High school/diploma/BA);
- How long have you been teaching? (Less than 5 years/between 5 and 10 years/more than 10 years);
- How old are you? (20–25 years/26–30 years/30–40 years); see Table 1.

Part 2 consisted of open-ended questions regarding the video cases. The case study analysis included a viewing of two 5-min videos of two children: one with ASD and the other with ADHD, both aged 6–8 years old. The child in video 1 had ASD; the participants viewed his interactions with his mother at home and during playtime and teaching time. Meanwhile, the child in video 2 had ADHD. The participants viewed the child in different situations, during class activities, at the swimming pool, and lunchtime. Then, the participants were asked to answer four questions:

- Is the child suffering from any problem? (Yes/No);
- “Based on your observation of the videos, what kind of disorder did the child have?” (Open response);
- “What are the reasons for your answer?” (Open response);
- “Based on your observation, would you refer the child’s parents to child intervention services? (Yes/No).

The skills of the preschool teachers at identifying children with ASD were measured based on two cases. The first case was an ASD case, for which permission to use the video was obtained from the Centers for Disease Control (CDC), USA. The other case was of an ADHD child, for which permission to use the video was obtained from the National Institute of Mental Health (NIMH). Previous studies also used this technique to help participants identify children with ASD [52,53,54]. These studies confirmed that observing children’s behaviour in their natural setting was the most ecologically valid method for assessing them [41]. It is also a good method to assess preschool teachers’ skills and their ability to identify children with ASD. 

Then, an expert panel consisting of four experts (one paediatric expert, one developmental psychologist, and two developmental specialists) validated the case study. Each expert had at least 5 years of experience in early ASD and childhood disorder identification and intervention. The panel’s rating for the case study was used as a standard to determine the correct answers. A high degree of inter-reliability was found among the experts’ scoring (intraclass correlation coefficient (ICC) = 0.79, 0.31–0.97 across Diagnostic and Statistical Manual of Mental Disorders, 5th Edition (DSM-5) criteria). Ten symptoms were determined for the ASD case and 10 symptoms for the ADHD case. The types of risk and urgency of referral were scored as the difference between the teachers’ answer and the average answer of the experts. The two open-ended questions were coded based on the number of signs and the type of information listed relative to the experts’ answers.

The open-ended questions were scored based on the number of signs specific to the ASD mentioned and the kind of disorder that the participants determined. Based on the categories that had been pre-defined in a content analysis procedure, the experts analysed 10 signs from preschool teachers (outside of this study). Each expert extracted the number and type of distinct ASD signs mentioned in the answers. The experts performed five rounds of discussions regarding disagreements until an 85% agreement was reached. In this study, the number of ASD signs and risk factors mentioned after watching the video cases was based on the presence of the categories coded in previous research.

### 2.4. Ethical Issue 

Ethical approval was sought from the University Putra Malaysia (UPM) and the reference number UPM/TNCPI/RMC/JKEUPM/1.4.18.2 (JKEUPM) was allocated as the approval for this project. As for the preschool teachers, they were informed that they had the right to withdraw from this study at any time or even to refuse to participate in the study.

### 2.5. Data Analysis 

In this study, a Mann–Whitney U Test was used to determine the preschool teachers’ ability to identify children with ASD by watching two videos (video 1 and video 2) of ASD and ADHD cases, based on their demographic variables (age, level of education, teaching experience). The preschool teachers’ responses to the questions related to the video cases were analysed using thematic analysis (AT). The data were determined through a rigorous process of data coding and development and revision. Data analysis was done manually by the researchers themselves because the responses of the participants were not so sophisticated that a software program was necessary.

## 3. Result

This study investigated Yemeni preschool teachers’ ability to identify children with ASD. The results of this study are presented in two sections. One section presents the quantitative findings including demographic data (the effects of age, education level, and teaching experience). The other section presents qualitative findings which were divided into four thematic concepts, as follows: preschool teachers’ skills at identifying the presence of behavioural problems, preschool teachers’ skills at naming the behavioural problem, preschool teachers’ skills at describing the behavioural problem, and preschool teachers’ recommendation to refer the child with ASD to a specialist. 

### 3.1. Quantitative Finding 

#### 3.1.1. Demographic Data 

The participants of this study were all female (20 teachers). They were divided into three age categories: 20–25 (30%), 26–30 (35%), and 31–40 (35%). Meanwhile, the teaching experience of preschool teachers were divided into three levels: <5 years (30%), between 5–10 years (50%), and >10 years (20%). Similarly, the preschool teachers’ education levels were also divided into three levels: high school level (30%), diploma level (50%), and degree level (45%). The profile of preschool teachers is presented in Table 1. 

#### 3.1.2. Effect of Age, Education Level, and Teaching Experience

To investigate the effect of the teachers’ age, education level, and teaching experience on their ability to identify children with ASD, this study used a Mann–Whitney Test. The test was validated and applied to the preschool teachers to determine their ability to identify children with ASD through two videos (video 1 and video 2), based on their demographic variables as it showed in Table 2. 

The Mann–Whitney test was conducted to investigate the interaction effect between preschool teachers’ demographic variables (age, educational level, teaching experience) on video 1 and 2. The results indicated that there was no statistically significant difference in the preschool teachers’ ability to determine if a child had behavioural problems (via observation of the video cases) regardless of their age, education level, and teaching experience.

### 3.2. Qualitative Finding

#### 3.2.1. Preschool Teachers’ Ability in Identifying the Presence of Behavioural Problem

All the preschool teachers (PST) (*n* = 20, 100%) were asked if they thought there was a problem with the children in the video cases. However, not all of the PST (30%, 10%) confirmed that the children had problems (video 1 and video 2, respectively) as it presented in Table 3. 

#### 3.2.2. Preschool Teachers’ Skills’ at Naming the Behavioural Problem

The preschool teachers’ explanation of the problem was also questioned. Table 4 shows a categorised list of the preschool teachers’ explanations of the child’s problem.

According to Table 4, thirty per cent (30%) of the preschool teachers (*n* = 6) expressed that there was no problem with the child in video 1, while for video 2, ten per cent (10%) of the preschool teachers (*n* = 2) confirmed that there was no problem with the child. They used phrases such as “I think he has no problem” “He is just spoilt” or “I think he has no problem, he just needs intensive attention from his teacher”.

The preschool teachers who answered, “Yes, there is a problem”, gave disparate views on the type of problems and the disorders of the children in the videos. 

On the other hand, twenty-five per cent (25%) of the preschool teachers (*n* = 5) determined that the child in video 1 was hyperactive and the same number (*n* = 5, 25%) of preschool teachers said that the child had autism. Similar to the situation in video 1, twenty-five per cent of the preschool teachers confirmed that the child in video 1 had a problem, an equal number answered that the child was hyperactive (*n* = 5), while twenty per cent (20%) of the preschool teachers (*n* = 4) said that the child had autism.

Next, one (*n* = 1, 5%) preschool teacher mentioned that the child in video 1 was spoilt/naughty and a few (*n* = 3, 15%) preschool teachers said that the child in video 2 was naughty and difficult to control.

Two answers from two teachers (*n* = 2, 10%) were unexpected, where both used the term ADHD to describe the child in video 1. The same percentage of preschool teachers (*n* = 2, 10%) said that the child in video 2 was feeling bored. Additionally, one preschool teacher (*n* = 1, 5%) supposed that the children in both videos had a kind of disorder, but most could not determine the exact disorder, so they used terms such as psychological disorder. Moreover, there were two (*n* = 2, 10%) preschool teachers who said that the child in video 2 lacked concentration, while another group of teachers (*n* = 2, 10%) said that the child was an introvert. 

#### 3.2.3. Preschool Teachers’ Ability in Describing the Behavioural Problem

The preschool teachers then gave examples to explain their answers. Thirty per cent (30%) considered the child in video 1 as not having any problems. The preschool teachers’ quotes are as follows: 

[PST4] “The child in the video has no problems. I have many children that behave like him.” 

[PST12] “I think he is just spoilt. He has no behavioural problem.” 

[PST14] “The child just needs more schooling from his parents and he has no problem.”

Meanwhile, 25% of the preschool teachers were confused between autism disorder and ADHD, as can be seen from their explanation of the problem of the child in video 1: 

[PST2] “He cannot express what he needs, so he throws things around.” 

[PST9] “I think he has autism because he plays by himself and he is hyperactive. Plus, he does not follow instructions, and he throws things around. He also cannot play correctly. He cannot focus on others’ eyes, and he does not deal well with others.” 

[PST10] “He does not make any eye contact and he repeats his words.” 

[PST18] “He stares at the ceiling fan for a long time, and he does not follow instructions.”

The above are the explanations the teachers gave to explain autism in video 1. To explain hyperactivity, the preschool teachers said: 

[PST15] “He is hyperactive; he wants to explore the world around him. Besides, he has a little bit of aggressive behaviour.”

[PST6] “He is jumping, he is moving his hands, and he is breaking things around him.”

[PST5] “He threw those things around and he is hyper-angry. He wants to explore the world around him, he jumps on things, he refuses to drink water, and he refuses to do his homework.”

[PST3] “He is hyperactive. He lacks concentration. He shows repetitive behaviours. The child cannot concentrate on anyone who talks to him, and he cannot control his movements. He has difficulties adapting with others and he hates annoying sounds but he is making those sounds.”

[PST1] “He plays with anything that is around him, he does not do his homework.”

The preschool teachers who said that a child had ADHD justified this by saying:

[PST12] “He cannot control his movements, so he is always moving and playing with anything he sees.”

[PST19] “This child is hyperactive and lacks concentration.”

The preschool teachers who expressed that the child was naughty in video 1, said that: 

[PST7] “He is naughty, and needs more schooling.”

One of the preschool teachers could not determine the type of disorder the child had, saying: 

[PST8] “He has a Psychological disorder, he plays with anything around him and pays no attention to anything else, and he does not follow instructions.” 

In video 2, the preschool teachers justified their answers as follows:

Some teachers said that the child was hyperactive, giving the following explanation:

[PST1] “The child is hyperactive. He is behaving strangely and he cannot concentrate except if someone holds him, like when his father held him in the video. He lives in his own world. He is autistic and introverted, he does not follow instructions, he does not like annoyances, and he becomes disturbed if he hears high-pitched sounds.”

[PST3] “He repeats his behaviours; he has learning disabilities.”

[PST6] “He is active and hates class activities.”

[PST8] “He plays alone far from other friends”

[PST10] “He does activities with himself; he does not care about his classmates. He needs a more engaging teaching strategy.”

[PST18] “He is active and loves to play most of the time.”

Meanwhile, [PST5] said that the child was hyperactive and displayed repetitive behaviours. She justified that, “He repeats his behaviours, so he has learning disabilities.” [PST9] said that the child “lacks concentration,” explaining that “He has no concentration and plays by himself.”

Some preschool teachers said that the child had autism, justifying their answer as follows:

[PST2] “He does not deal with other classmates. He does not join in with others in sing-along activities. He imitates other friends to mimic their behaviour. He does not like annoyances. He does not like his food.”

[PST5] “He plays alone far from other friends.”

[PST7] “He lives in his own world, and he flaps his hands.”

[PST19] “He does not pay any attention to his teacher; he prefers to play alone.”

Meanwhile, the preschool teachers that expressed that the child in video 2 was an introvert explained their answer as follows: 

[PST14] “He does not share with his classmates. He does not follow instructions, and he is annoyed with high-pitched sounds.”

[PST15] “This child does not love to share with other classmates.”

In addition, some of the preschool teachers though that the child in video 2 was naughty, and they explained their reasoning as follows: 

[PST12] “The child is just naughty; he needs his teacher to control his behaviour.”

[PST13] “The child’s parents should control his behaviour.”

[PST16] “I think he is spoilt.”

Lastly, one preschool teacher [PST5] expressed that the child felt bored. She justified her answer as follows: “He just felt bored with the school activities so that is why he stopped paying attention to his teachers”.

#### 3.2.4. Preschool Teachers’ Recommendations to Refer the Child with ASD to a Specialist 

When the preschool teachers were asked whether or not they would refer to the child’s parents to a specialist, different answers were given, as shown in Table 5.

According to Table 5, 75% of the preschool teachers (*n* = 15) answered that the child in video 1 (who had autism spectrum disorder) did not need to be referred to a specialist. Meanwhile, 25% of the preschool teachers (*n* = 5) believed that the child had to be referred. In contrast, the preschool teachers’ opinions on the child in video 2 (who has ADHD) were significantly different than their opinions on the child in video 1. In total, 60% of the preschool teachers (*n* = 12) confirmed that the child in video 2 had to be referred to a specialist and that his parents should be told to do so. On the other hand, 40% of the preschool teachers (*n* = 8) confirmed that the child with ADHD in video 2 did not need a referral. 

## 4. Discussion

This study evaluated the teachers’ ability to accurately identify preschool-age children with ASD, using video cases of children with developmental delay. Schools are now considered to play a vital role in the identification and management of children’s mental health, yet few studies have examined preschool teachers’ ability to identify children with symptoms of autism spectrum disorder (ASD). However, preschools have been identified as ideal settings for identifying children with potential developmental delays, such as ASD, and so teachers must work with parents to access special services because the former work with many children every day and may have intimate knowledge of a child’s development [55,56]. 

The first objective of this study was to determine preschool teachers’ ability to detect the behavioural problems of the children presented in each of the video cases. The results showed that the preschool teachers’ had a moderate ability to detect behavioural problems in the children, even though the symptoms of behavioural disorders such as autism, attention deficit hyperactivity disorder (ADHD), depression, and anxiety can be detected in children before they begin elementary school [17,19,57]. In this study, it was found that some of the preschool teachers could not determine if the children in the videos had any behavioural problems. They felt that the children were spoilt or that the children’s parents did not educate them properly. These findings confirmed the findings of Shamsudin (2014) [58] that, unfortunately, the public is not very well educated about autism. Many of them perceive ASD children as spoilt children. Meanwhile, the parents of these children are seen as parents who cannot control their offspring, with some even being considered immature parents with misbehaving children. In other words, not all preschool teachers are familiar with identifying children with external problems such as ADHD or internal problems and emotional problems such as ASD, as confirmed by Neil [59], who mentioned that preschool teachers only have a modest sensitivity towards children’s internalised symptoms.

The second objective of this study was to explain preschool teachers’ ability to recognise a child with ASD. It was found that the preschool teachers gave varied answers. Some of them mistakenly identified the symptoms of ADHD as the symptoms of ASD. The same finding was found in previous research, where teachers also mistakenly identified symptoms of ADHD as symptoms of ASD [29,46]. Johnson et al. (2012) [60] included ADHD symptoms on a scale to test the ability of study participants to distinguish the symptoms of the two childhood disorders. The finding confirmed that preschool teachers mistakenly agreed that the symptoms of ADHD were also the symptoms of ASD, hence proving that the teachers could recognise hallmark red flags of childhood disorders, but that it was difficult for them to distinguish between the two disorders. This case is understandable because, even for a trained professional, the symptoms of one disorder could sometimes overlap and be mistaken for another. Preschool teachers can and should, therefore, be considered a reliable source for making referrals for further assessments, but they should be trained on how to more accurately distinguish childhood disorders and normal childhood development. Additionally, preschool teachers should be provided with more training to enhance their ability to recognise the early signs of ASD in young children. Therefore, the present research did not find any significant results linking particular factors to the teacher’s ability to recognise the symptoms of ASD. This study hypothesised that those who had more experience working in the field of early childhood education would have a greater ability to recognise the symptoms of ASD in young children. However, this hypothesis was not supported, as there was no difference between the ASD recognition scores of those who had more years of teaching experience and those who had fewer years of experience. This result suggests that the teachers’ experience working in the field of early childhood education did not impact their ability to recognise the symptoms of ASD in young children. Future research should, therefore, examine whether or not there is a difference between the ability to recognise the symptoms of ASD between teachers who have experience working in preschool settings, which claim to be inclusive, and those who have experience working in preschool settings, which are ill-equipped for teaching students with greater needs due to disabilities.

Thirdly, the purpose of this study was to address teachers’ readiness to refer a child to specialists. Early detection of such problems and subsequent referral to appropriate services can alter a child’s trajectories and prevent more serious problems from developing. However, research has shown that although preschool teachers can identify socio-emotional problems in their students, there are often gaps in their knowledge regarding behavioural and emotional problems. They also showed variability in comfort and confidence regarding their role of problem identification and referral, resulting in limited identification and referral skills for these types of problems [40,41]. Interestingly, most of the preschool teachers recommended referral for children with ADHD, meaning that children with external problems would more likely get help, possibly because preschool teachers tend to evade their responsibility to deal with a child that shows internal behavioural problems. On the other hand, preschool teachers do not tend to refer children with ASD to specialists, as these children stay quiet in class most of the time.

Subsequently, the final objective of this study was to determine the effect of demographic variables (age, education level, and teaching experience) on preschool teachers’ ability to identify children with ASD. This study did not find any significant result that link particular factors to preschool teachers’ ability to recognise the symptoms of ASD. This study hypothesised that teachers who had a higher education level would have a greater ability to identify children with ASD. This hypothesis was not supported; however, teachers with a high education level and teachers with a lower education level showed no difference in their ability to identify ASD in this study. In the context of Yemen, most Yemeni preschool teachers did not graduate from the childhood education field or kindergarten department and sometimes Yemeni preschool teachers only graduated from other Departments to become the secretary of that department. 

On the other hand, in this study, the teaching experience of preschool teachers was not correlated to their ability to identify children with ASD. Moreover, the hypothesis that more teaching experience correlated to a better ability to identify children with ASD was not supported, as those who had more years of teaching experience and those who had fewer years of teaching experience did not show differences in the ability to identify ASD. The reason for this case is that Yemeni preschool teachers, as mentioned before, did not graduate with a degree in childhood development, which means that their work experience might not be linked to childhood development or teaching children. The results of the present study also agree with that of Drusch (2015), who confirmed that the working experience among preschool teachers was not linked to their ability to recognise ASD in children. 

The result of this study suggests that teachers’ training and professional development may be an effective way to target the identified factors that influence teachers’ referral decisions. Training could improve teachers’ referral rate. In another study, teachers gave different reasons for referring children with excessive anxiety [36]. In this case, training could focus on equipping teachers with a decision-making model that will guide them through the most appropriate reasons for deciding to make a referral. Such a model will outline appropriate tipping points to achieve an optimal threshold for referral. 

This study had some limitations. Firstly, the sample size was small, which could impact the result. Moreover, the study focused on the preschool teacher’s ability or identification skills and not the knowledge of preschool teachers regarding ASD. Therefore, future work should expand on the knowledge and identification skills of teachers regarding ASD. Additionally, this study focused on mainstream preschool teachers. Therefore, we suggest that future studies should compare mainstream preschool teachers with special needs preschool teachers. 

## 5. Conclusions

The Yemen Education Development Plan (2014–2018) has highlighted special education as a focus under the rights to education. Hence, various efforts have been undertaken to uplift special education in the educational call for all. However, there is still room to refine this area, especially areas that involve disclosure and training to primary teachers in special education, because there is still a large gap between the knowledge and understanding of primary teachers regarding special education in general. Hence, the cooperation of all parties, education ministries, policy implementers, parents, and the community, is essential to realise the educational revolution that would raise the dignity of special education. This research is important to the field of social work because it is imperative to ensure that public agencies are working to help families gain access to services that will aid their children to reach their full potential. It is important to provide inclusive classroom settings for young children with ASD so that these children will be able to learn and gain the same opportunities through play with peers and adults. Moving forward, social workers should better inform preschools about the diverse needs of students with developmental delays, such as ASD. Social workers should teach preschools to recognise the signs of ASD, provide tips for engaging in conversations about concerns with caregivers and offer supports related to incorporating mainstream best practices into preschool settings. To this end, social workers should reach out to preschools and offer to provide education and support for their staff. It is also important to keep in mind that families of children who have the most severe forms of ASD may not be satisfied with regular preschool settings. Social workers can provide parents with other options for schools that specifically cater to children with ASD and other high-needs disabilities. Social workers can also work to provide these resources to parents, as well as to preschool centres so that the preschool centres can refer the children to programs that may better meet their needs.

## Figures and Tables

**Table 1:** Respondent profile.

Demographic Variables	Rank	f	%
Age	20–25	6	30
	26–30	7	35
	31–40	7	35
Education level	High school	4	20
	Diploma	7	35
	Bachelor	9	45
Teaching experience	<5	6	30
	5–10	10	50
	>10	4	20

**Table 2:** The effect of demographic variables in video 1 and video 2.

Age		Groups	*N*	Mean Rank	Sum of Ranks	Significant
20–25	identify1	Video 1	6	6.50	39.00	1.000
		Video 2	6	6.50	39.00	
		Total	12			
26–30	identify1	Video 1	7	7.00	49.00	0.606
		Video 2	7	8.00	56.00	
		Total	14			
31–40	identify1	Video 1	7	7.50	52.50	1.000
		Video 2	7	7.50	52.50	
		Total	14			
**Education**		**Group**	***N***	**Mean Rank**	**Sum of Ranks**	**Sig**
high school	identify1	Video 1	4	4.50	18.00	1.000
		Video 2	4	4.50	18.00	
		Total	8			
diploma	identify1	Video 1	7	7.50	52.50	1.000
		Video 2	7	7.50	52.50	
		Total	14			
bachelor	identify1	Video 1	9	9.00	81.00	0.609
		Video 2	9	10.00	90.00	
		Total	18			
**Teaching Experience**		**Group**	***N***	**Mean Rank**	**Sum of Ranks**	**Sig**
>5	identify1	Video 1	6	6.50	39.00	1.000
		Video 2	6	6.50	39.00	
		Total	12			
5–10	identify1	Video 1	10	10.00	100.00	0.661
		Video 2	10	11.00	110.00	
		Total	20			
<10	identify1	Video 1	4	4.50	18.00	1.000
		Video 2	4	4.50	18.00	
		Total	8			

**Table 3:** Preschool teachers’ rate in identifying the presence of behavioural problem.

	Video 1	Rate	Video 2	Rate
1	Hyperactive	5	Hyperactive	5
2	Autism	5	Autism	4
3	Psychological disorder	1	Lack of concentration	2
4	ADHD	2	Introvert	2
5	Spoilt	1	Boring	2
6	No problem	6	Spoilt/naughty	3
			No problem	2
	Total	20	Total	20

**Table 4:** Preschool teachers’ ability in naming the behavioural problem.

	Responses	Video 1	Video 2
1.	PST 1	hyperactive	Hyperactive and autism
2.	PST 2	Autism	Autism
3.	PST 3	hyperactive	Hyperactive and repeated behaviours
4.	PST 4	No	I think he has no problem
5.	PST 5	Hyperactive	Autism
6.	PST 6	Hyperactive	Hyperactive
7.	PST 7	Spoilt	Naughty
8.	PST8	Psychological disorder	Autism
9.	PST9	Autism	Lack of concentration
10.	PST10	Autism	Hyperactive
11.	PST11	No Problem	Boring
12.	PST12	ADHD	Boring
13.	PST13	No Problem	Naughty
14.	PST14	No problem	Introvert
15.	PST15	hyperactive	Introvert
16.	PST16	No problem	Spoilt
17.	PST17	Autism	Lack of concentration
18.	PST18	Autism	Hyperactive
19.	PST19	ADHD	Autism
20.	PST20	No Problem	Normal child

**Table 5:** Child referral decision.

Refer Decision	Yes		No	
	f	%	f	%
**Video 1**	5	25	15	75
**Video 2**	12	60	8	40
